# Mapping the pH Sensors Critical for Host Cell Entry by a Complex Nonenveloped Virus

**DOI:** 10.1128/JVI.01897-18

**Published:** 2019-02-05

**Authors:** Weining Wu, Cristina C. Celma, Adeline Kerviel, Polly Roy

**Affiliations:** a Department of Pathogen Molecular Biology, Faculty of Infectious and Tropical Diseases, London School of Hygiene and Tropical Medicine, London, United Kingdom; Loyola University Medical Center

**Keywords:** BTV, entry, histidine, pH sensor, zinc finger

## Abstract

Bluetongue virus (BTV), in the family Reoviridae, is an insect-borne, double-capsid virus causing hemorrhagic disease in livestock around the world. Here, we elucidate how outer capsid proteins VP2 and VP5 coordinate cell entry of BTV. To identify key functional residues, we used atomic-level structural data to guide mutagenesis of VP2 and VP5 and a series of biological and biochemical approaches, including site-directed mutagenesis, reverse genetics-based virus recovery, expression and characterization of individual recombinant mutant proteins, and various *in vitro* and *in vivo* assays. We demonstrate the dynamic nature of the conformational change process, revealing that a unique zinc finger (CCCH) in VP2 acts as the major low pH sensor, coordinating VP2 detachment, subsequently allowing VP5 to sense low pH via specific histidine residues at key positions. We show that single substitution of only certain histidine residues has a lethal effect, indicating that the location of histidine in VP5 is critical to inducing changes in VP5 conformation that facilitates membrane penetration. Further, we show that the VP5 anchoring domain alone recapitulates sensing of low pH. Our data reveal a novel, multiconformational process that overcomes entry barriers faced by this multicapsid nonenveloped virus.

**IMPORTANCE** Virus entry into a susceptible cell is the first step of infection and a significant point at which infection can be prevented. To enter effectively, viruses must sense the cellular environment and, when appropriate, initiate a series of changes that eventually jettison the protective shell and deposit virus genes into the cytoplasm. Many viruses sense pH, but how this happens and the events that follow are often poorly understood. Here, we address this question for a large multilayered bluetongue virus. We show key residues in outer capsid proteins, a pH-sensing histidine of a zinc finger within the receptor-binding VP2 protein, and certain histidine residues in the membrane-penetrating VP5 protein that detect cellular pH, leading to irreversible changes and propel the virus through the cell membrane. Our data reveal a novel mechanism of cell entry for a nonenveloped virus and highlight mechanisms which may also be used by other viruses.

## Abstract (precis)

Virus entry into a susceptible cell is the first step of infection and a significant point at which infection can be prevented. To enter effectively, viruses must sense the cellular environment and, when appropriate, initiate a series of changes that eventually jettison the protective shell and deposit virus genes into the cytoplasm. Many viruses sense pH, but how this happens and the events that follow are often poorly understood. Here, we address this question for a large multilayered bluetongue virus. We show key residues in outer capsid proteins, a pH-sensing histidine of a zinc finger within the receptor-binding VP2 protein, and certain histidine residues in the membrane-penetrating VP5 protein that detect cellular pH, leading to irreversible changes and propel the virus through the cell membrane. Our data reveal a novel mechanism of cell entry for a nonenveloped virus and highlight mechanisms which may also be used by other viruses.

## INTRODUCTION

The host cell entry mechanism of viruses, particularly large (>500 Å in diameter), nonenveloped capsid viruses, is a highly coordinated process, often engaging more than one viral protein with multiple conformational stages. Unlike enveloped viruses and smaller nonenveloped viruses, the atomic details of these processes for large, complex, nonenveloped viruses are largely unknown. Recently, we determined the structure of bluetongue virus (BTV), an 85-nm triple-layered complex capsid virus at atomic resolution, revealing key features of the outer capsid proteins that may facilitate the viral entry process (1).

BTV, a member of the Orbivirus genus of the family Reoviridae, is an agriculturally and economically significant insect-borne virus that causes serious illness and death in sheep and other domestic and wild ruminants in many parts of the world. Infection of mammalian cells by the BTV particle is established when the two-layered inner capsid, the “core” of the double-capsid particle, is translocated across the endosomal membrane following virus uptake (2). The two outer capsid proteins VP2 and VP5 are responsible for this process (3, 4). The larger VP2 protein (110 kDa) binds to the surface of the cells and facilitates clathrin-mediated endocytosis of the virion, while the smaller VP5 protein (60 kDa) is believed to penetrate the host cell membrane and deliver the 75-nm core particle into the host cytosol (5, 6). During this two-stage process, VP2 senses the pH (6.0 to 6.5) in the early endosome and detaches itself (1) from the remaining particle, which then proceeds to the late endosome where VP5 senses the lower pH (∼5.5) of the late endosome and undergoes significant a conformational change. The altered form of VP5 interacts with the membrane and causes membrane destabilization (“fusion” activity), permitting the core to escape into the cytoplasm. However, the molecular details of this process, in particular the coordination of the two outer capsid proteins, are unclear, in part due to the historical lack of atomic detail. The final product of the disassembly of BTV and of all members of the Reoviridae family is the transcriptionally active double-layered particle able to initiate transcription of the genomic RNAs. The two outer capsid proteins of BTV are supported by the surface layer of this double-layered particle or core, formed by 260 trimers of VP7, which coats the internal VP3 layer (7, 8). The viral transcriptase complex of three proteins VP1, VP4, and VP6 and the 10 genomic double-stranded RNA (dsRNA) segments (S1 to S10) are encapsidated by the VP3 layer.

The high-resolution (3.5-Å) structure of BTV, obtained by cryo-electron microscopy, revealed an outer shell formed by 120 globular trimers of VP5 and 60 triskelion-like VP2 trimers (1). The 961 residues of VP2 monomer are segregated into four domains, namely a hub domain that consists of both amino and carboxyl terminus (M1-Y49, G121-C162, and K839-V961), a body domain with most of the remaining middle region (L163-K190 and Y408-T838) and extends to a highly flexible external tip domain, and a small hairpin domain (D50-V120) between the hub and body domains. A typical zinc finger motif, a CCCH tetrahedron, is found between the interface of the hub and body domains (1).

The 526 residues of VP5 fold into three distinct domains, namely, dagger (M1-S68), unfurling (K69-F354), and anchoring (I355-A526). The unfurling domain is helix rich, with two long horizontal helices and a stem helix. Two parallel β strands connect the unfurling domain with the anchoring domain via a third antiparallel β strand. The anchoring domain has a cluster of histidine located within the four antiparallel β strands, and an N-terminal β strand tethers the dagger domain. Previous data demonstrated that VP2 detaches from the BTV particle when treated with acidic pH and VP5 undergoes conformational change (1). Further, recombinant VP5 could penetrate cellular membranes following low pH treatment (6, 9). However, the molecular mechanism by which VP2 and VP5 sense acidic pH during virus entry remains unknown.

To elucidate the molecular mechanisms by which VP2 and VP5 coordinate BTV entry, we used atomic-level structural data to inform a series of structure-guided substitution mutations in VP2 and VP5, followed by biochemical analyses of the mutant proteins *in vitro* and *in vivo* virus replication by reverse genetics. Together, these data revealed a novel entry mechanism for BTV not seen to date by other members of the Reoviridae in which the VP2 zinc finger senses the low pH of the early endosome and VP5 senses the late endosomal low pH, resulting in coordinated changes to protein conformation, which, in turn, facilitate membrane penetration. This comprehensive molecular and biochemical analysis, which complements our atomic-level structural data, reveals a novel mechanism of cell entry by a complex, nonenveloped virus and provides mechanisms that may be shared with other capsid viruses.

## RESULTS

### Mapping pH-sensing histidine residues in VP2 and their importance in virus replication.

**FIG 1:**
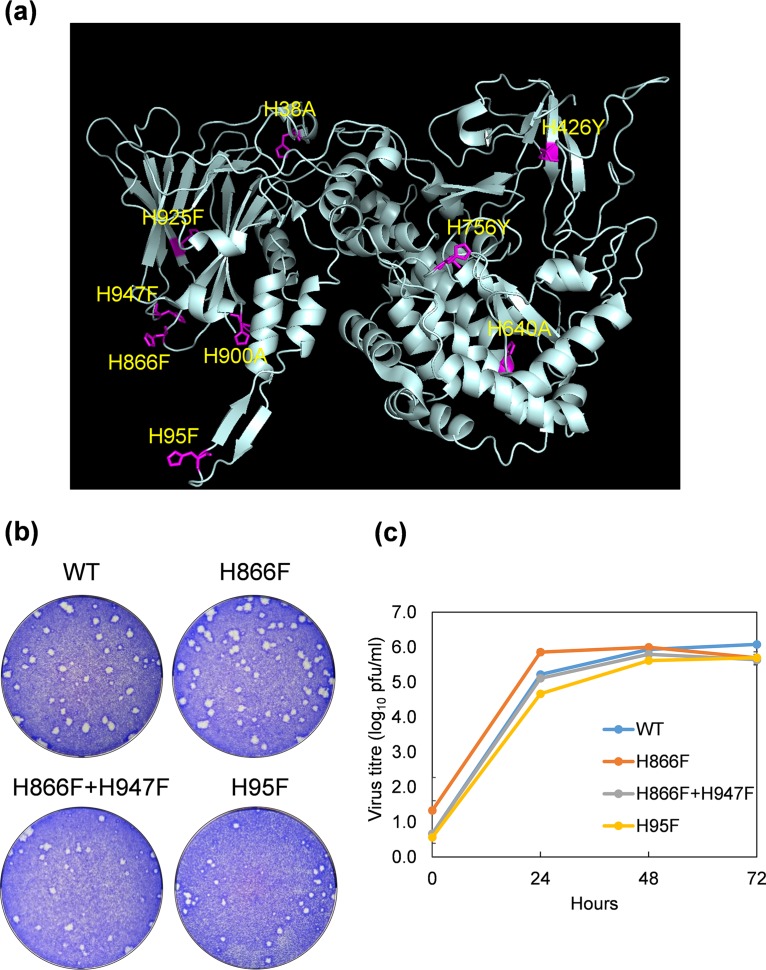
His residues in VP2 targeted for mutagenesis. (a) Mutations introduced to all other conserved His residues (except for H164 within the zinc finger motif) in BTV1 VP2 which might be involved in sensing early endosome low pH. H866, H947, and H95 predicted previously, based on structural analysis and others chosen by sequence alignment of different BTV serotypes (see Fig. S1 in the supplemental material). (b) Plaque assay showing the phenotype of H866F, H866F+H947F, and H95F mutant viruses compared with that of the wild-type virus. (c) Single-step viral growth curve of H866F, H866F+H947F, and H95F mutant viruses compared with that of the wild-type virus.

Histidine residues are known to play a key role in sensing pH by protonation in many cases of virus entry, such as in the influenza hemagglutinin (HA) protein (10) and the alphavirus and flavivirus fusion proteins (11). The VP2 of BTV serotype 1 (BTV1) possesses 28 His residues; several of these His residues are highly conserved among all 25 known BTV serotypes, indicating they may play an important functional role during virus entry (1). VP2 is known to be detached from virions in the early endosome (12), and based on its high-resolution structure (1), we hypothesized that several His residues could form part of a pH-sensing mechanism. The hub domains of three VP2 monomers, each with nine His residues, interact to form a VP2 trimer, which sits atop four VP7 trimers on the underlying core surface. Two conserved His residues, H866 and H947, are located at the VP2-VP7 interface and would be consistent with the pH-sensing role. Similarly, structural data showed H95 in the hairpin domain interacts with the VP5 layer and is likely to play a role in VP2 detachment. Six other His residues which are highly conserved (see Fig. S1 in the supplemental material) could also fulfill this function. Three of these are located in the hub domain, H38 at a β sheet on the surface of the domain, while H900 and H925 are at the base of the domain. Other three residues are dispersed in the body domain, namely, H426 at the β-sheet-rich apex of the domain, which may be involved in the interaction with the VP2 tip domain, and H640 and H756 located in a α-helix-rich base within the body domain (Table 1; Fig. 1a). Each of these residues was targeted for mutagenesis, substituting for either alanine, phenylalanine, or tyrosine, and the mutated VP2 RNA molecule was included in a reverse genetics (RG) system to allow the recovery of BTV carrying each mutant variant of VP2 (Table 1).

**TABLE 1:** Mutations introduced to BTV1 VP2

Mutation	Reverse genetics	Virus growth in BSR cells	Recombinant protein
Hub domain			
Zinc finger			
H164C	Not recovered	NA^*a*^	Expression severely compromised
H164F	Not recovered	NA	ND^*b*^
C162H	Not recovered	NA	Expression compromised, not binding to zinc, not sensing low pH
C617H	Not recovered	NA	ND
C851H	Not recovered	NA	ND
C162H+H164C	Not recovered	NA	ND
C617H+H164C	Not recovered	NA	ND
C851H+H164C	Not recovered	NA	ND
H866F	Recovered	Similar to WT	ND
H866F+H947F	Recovered	Similar to WT	ND
H38A	Recovered	Similar to WT	ND
H640A	Recovered	Attenuated	ND
H900A	Recovered	Similar to WT	ND
Hairpin domain			
H95F	Recovered	Similar to WT	ND
Body domain			
H426Y	Recovered	Similar to WT	ND
H756Y	Recovered	Similar to WT	ND
H925F	Recovered	Similar to WT	ND

a NA, not applicable.

b ND, no data.

**FIG 2:**
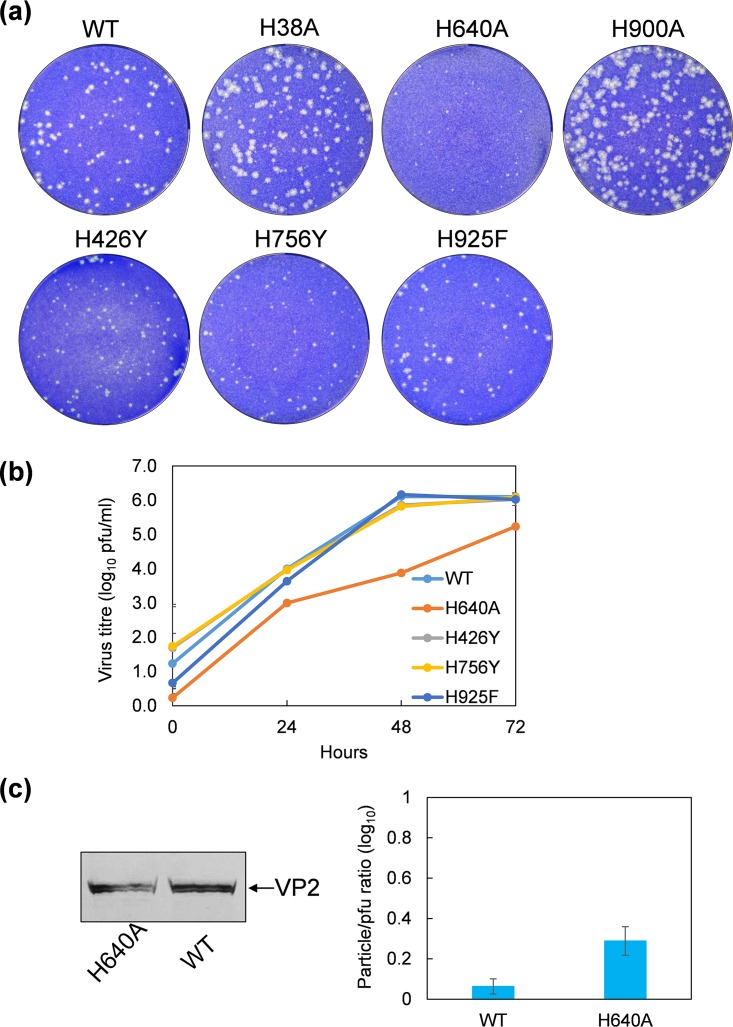
Phenotype and growth kinetics of VP2 mutant viruses. (a) Plaque assay of H38A, H640A, H900A, H426Y, H756Y, and H925F mutant viruses compared with wild-type virus. (b) Single-step viral growth curve of H640A, H426Y, H756Y, and H925F mutant viruses compared with that of the wild-type virus. (c) Analysis of the expression of H640A mutant protein compared with the wild-type VP2 in transfected BSR cells by Western blot using a VP2 antibody (left). Average particle/PFU ratios of wild-type and H640A mutant viruses are 1.2 and 2.0, respectively (right).

Surprisingly, all mutants could be recovered by reverse genetics with very similar phenotypes to the wild-type virus except H640A, which showed marginal attenuation (Fig. 1b and 2a). Growth curves of each of VP2 mutant virus confirmed that only the H640A mutation had a modest effect on virus growth (Fig. 1c and 2b). Since residue H640 lies in the structurally stable region away from the interfaces, it is, therefore, unlikely to play a major role in sensing cellular low pH, although it may also contribute to the pH sensing. To ensure that the H640 mutation did not perturb protein expression generally, BSR cells were transfected with a H640A mutant VP2 plasmid, and the expression of VP2 was analyzed by Western blot using a polyclonal VP2 antibody. The Western blot analysis showed the mutant VP2 expression was equivalent to the wild-type protein, indicating that the H640A mutation had no significant effect on VP2 expression (Fig. 2c, left). In addition, we estimated the average particle/PFU ratio of the H640A mutant virus. The number of particles on the basis of a viral genome copy number determined by reverse transcription-quantitative PCR (qRT-PCR) versus PFU, which was approximately 2.0, was not statistically different than the wild-type virus, with an average particle/PFU ratio of 1.2 (Fig. 2c, right), suggesting that this mutation did not significantly alter the efficiency of virus production.

Our overall data indicate that none of the His residues that were targeted for mutagenesis analysis are critical for VP2 function as a virus entry mediator; therefore, the major pH sensor required for VP2 conformational change in the endosomal compartment must lie elsewhere.

### The single histidine residue in the VP2 zinc finger and its correct position at the tetrahedron is vital for virus entry.

**FIG 3:**
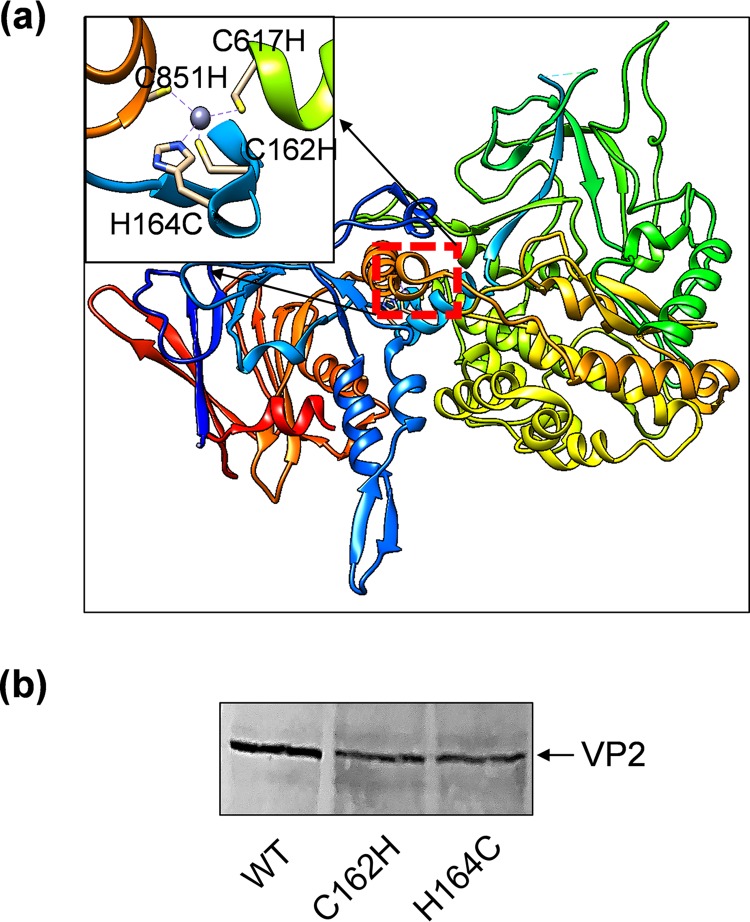
Detailed structure of the highly conserved tetrahedron zinc-finger motif formed by residues C162, C617, C851, and H164. (a) Single mutation C to H or H to C was introduced to each residue individually (highlighted in the top left box), or double mutations C162H+H164C, C617H+H164C, and C851H+H164C were introduced to alter the arrangement of the zinc finger but the integrity of the CCCH cluster remained. (b) Analysis of the expression of C162H and H164C mutant VP2 proteins compared with the wild-type VP2 in transfected BSR cells by Western blot using a VP2 antibody.

The high-resolution structure of VP2 identified a typical zinc-finger motif at the junction of the body and the hub domains of each monomer, formed by residues C162, C617, C851, and H164, which is highly conserved among all 25 BTV serotypes (1). Structural data suggested the zinc finger may function to maintain the VP2 in a metastable state and may participate in the detachment of VP2 in low pH in concert with H866, which was predicted to be the key residue for sensing low pH and disruption of VP2 and VP7 interaction. Since the above data showed that H866A had no apparent effect in virus recovery, we investigated whether or not CCCH could act as a pH-sensing switch for VP2 detachment, as protonation of His164 would be expected in the acidic conditions of the endosome. To do this, we introduced a single substitution mutation to mutate the highly conserved H164 to Cys (Fig. 3a). In contrast to the nine His mutations discussed above, no virus recovery was observed with this H164C mutant. This effect was not due to a disruption of mutant protein expression, since Western blot analysis showed that the H164C mutant was expressed in BSR cells when transfected with the mutant plasmid, albeit at slightly reduced level (Fig. 3b). To rule out the possibility that the cysteine substitution might have affected protein folding, H164 was further substituted with phenylalanine, and the H164F mutant was used for virus recovery in the RG system. As with the H164C mutant, however, no virus was recovered, suggesting that a histidine at this position is critical for virus fitness and that its substitution with any alternative residues is not tolerated for VP2 function.

Furthermore, when the three cysteines of the finger, C162, C617, and C851, each substituted by His to compensate for the H164C substitution in the vicinity, none was successfully rescued as live virus using our RG system (Fig. 3a). This effect was also not due to the disruption of mutant protein expression, since Western blot analysis showed that the C612H mutant was expressed in BSR cells when transfected with the mutant plasmid, although at slightly reduced levels (Fig. 3b). This suggests that His164 is an essential position for VP2 function, which cannot be substituted by the provision of other His residues within the zinc-finger motif. To investigate this further, we altered the arrangement of the zinc finger by swapping C162H+H164C, C617H+H164C, and C851H+H164C, maintaining the integrity of the CCCH cluster but scrambling its order (Fig. 3a; Table 1). None of these mutants was rescuable as virus by the RG system, indicating that the parental CCCH cluster is an essential component of VP2 function. Previously we showed, using recombinant VP2 *in vitro*, that chelation of zinc led to VP2 instability manifested by altered properties of thermal denaturation (1). Thus, our observations *in vivo* would be consistent with the protonation of His164 within the zinc-finger motif located at the interface of body and hub domains, leading to loss of zinc coordination and VP2 conformational change.

### Analysis of recombinant zinc-finger mutant proteins confirms the zinc-finger motif is involved in a pH-sensing conformational change of VP2.

**FIG 4:**
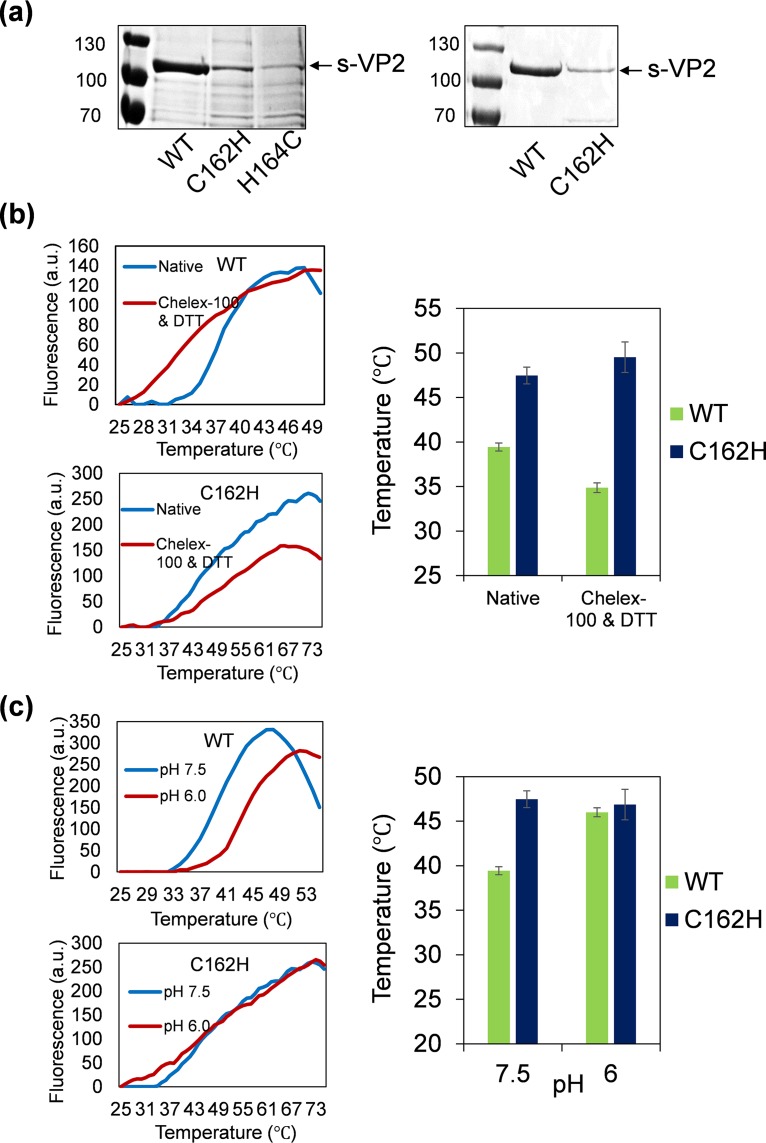
Analysis of recombinant VP2 mutant proteins. (a) Expression level of wild-type or C162H and H164C mutant s-tagged VP2 protein in whole-cell lysate of recombinant baculovirus-infected sf9 cells examined by SDS-PAGE with Coomassie blue staining (left). Purified wild-type and C162H mutant s-tagged VP2 proteins confirmed by SDS-PAGE with Coomassie blue staining (right). (b) Effect of zinc metal on the thermal stability of wild-type or C162H mutant protein was measured by thermal shift assay with or without treatment of Chelex-100 metal chelating resin and DTT reducing agent. (c) Similar to (b), the effect of pH on the stability of wild-type and C162H mutant protein was measured. In both conditions, the denaturation midpoint melting temperature (*T_m_*) of wild-type and C162H mutant protein was compared and presented as histograms, and corresponding melting curves are presented in the left panels.

**FIG 5:**
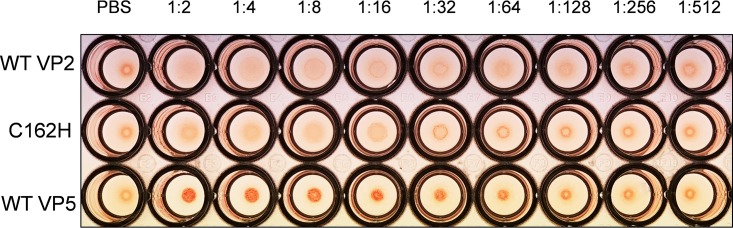
Hemagglutination activities of wild-type and C162H mutant VP2 proteins were assessed by hemagglutination assay with sheep erythrocytes. Wild-type VP5 protein was used as a negative control.

Based on our previous observation that zinc chelation led to VP2 instability (1), we investigated whether this may explain the inability to recover viruses in the RG system. To test this, we expressed two of the nonrecoverable VP2 mutants, C162H and H164C, using the baculovirus expression system and purified them. The expression level of both proteins and particularly H164C was lower than the wild-type protein (Fig. 4a, left), suggesting a degree of instability. However, VP2 C162H could still be purified sufficiently for a thermal shift stability assay (Fig. 4a, right). Accordingly, VP2 C162H was treated with Chelex-100 to remove bound divalent zinc ion in the presence of the reducing agent dithiothreitol (DTT) to prevent the formation of a disulfide bond between the remaining cysteines of the CCCH motif after removal of the zinc ion, and its thermal shift profile was compared with that of the wild-type (WT) VP2. In contrast to the distinct shift of the melting curve and substantial reduction in the melting temperature of the wild-type protein associated with Zn chelation, no significant change with the melting curve and temperature were observed for the mutant protein (Fig. 4b). Furthermore, when the assay was repeated with an altered pH in place of the thermal shift, wild-type VP2 similarly underwent substantial changes of the melting curve and temperature when the pH was shifted from neutral pH 7.5 to early endosomal pH 6.0, while VP2 C162H did not (Fig. 4c). Although the C162H VP2 mutant failed to sense the low pH, it should still retain its ability to attach to the cell surface. Since VP2 is responsible for hemagglutination (3), a hemagglutination assay was performed using sheep erythrocytes. The result demonstrated that VP2 C162H retained the hemagglutinating activity, similar to that of wild-type VP2, and in contrast to VP5, which did not show any hemagglutinating activity (Fig. 5), indicating that C162H mutation did not affect the attachment of VP2 to blood cells. Taken together, these data confirm that the unique zinc-finger motif within VP2 is the pH-sensing element for conformational change in the early endosome. Our data demonstrate that for BTV VP2, the CCCH motif functions as the sole pH-sensing element and cannot be replaced by the multiple His residues throughout the protein. This is consistent with an abrupt conformational transition necessary to reveal VP5 soon after virus entry.

### Determination of the importance of histidine clusters in the membrane penetration protein VP5.

**FIG 6:**
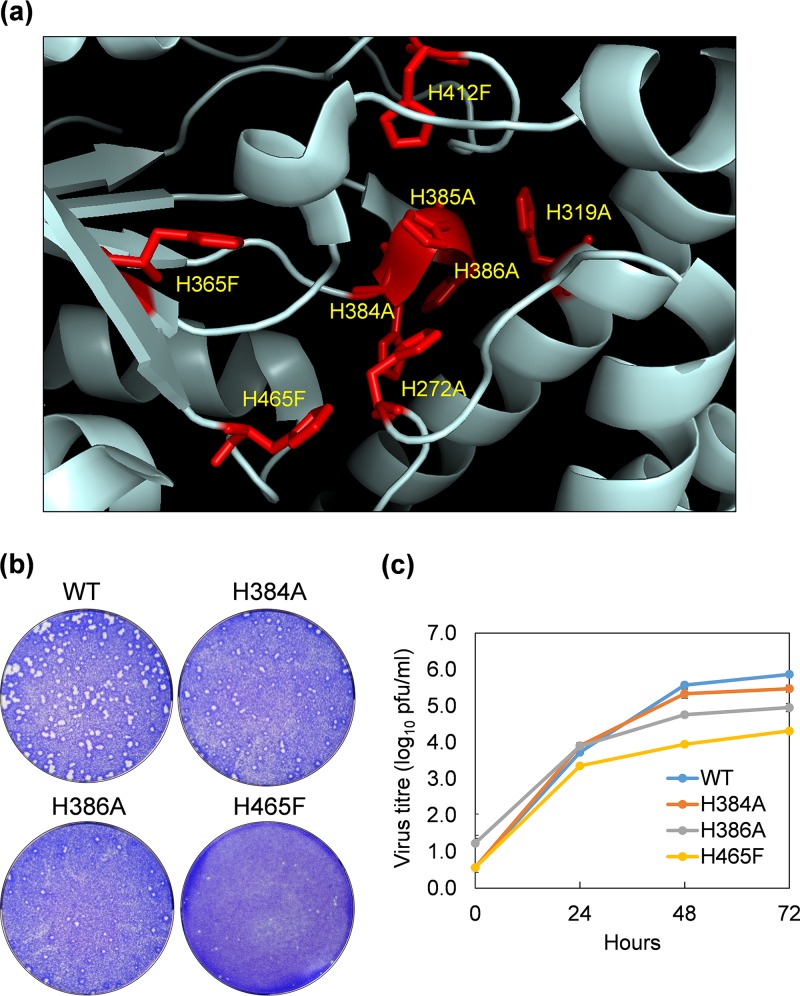
His residues in VP5 targeted for mutagenesis. (a) Mutations introduced to the His cluster at the interface between the β-meander motif of the anchoring domain (H365F, H384A, H385A, H386A, H412F, and H465F) and the beam helices of the unfurling domain (H272A and H319A) in BTV1 VP5 based on structural analysis, predicted to be involved in sensing late endosome low pH. (b) Plaque assay showing the phenotype of H384A, H386A, and H465F mutant viruses compared with that of the wild-type virus. (c) Single-step viral growth curve of H384A, H386A, and H465F mutant viruses compared with that of the wild-type virus.

Since, in BTV infected cells, virion particles lacking VP2 traffic from the early- to late-endosomal compartment, VP5 must sense late-endosomal pH prior to interaction with the endosomal membrane. VP5 is rich in His residues, mainly dispersed in two domains, the unfurling domain (UNF) and the anchoring domain (ANC) (1). Many are clustered closely at the interface between the β-meander motif of the ANC and the beam helices of UNF, and these could be responsible for sensing the acidic pH of the late-endosomal compartment (Fig. S2). Thus, eight His residues, H272, H319, H365, H384, H385, H386, H412, and H465, located in the interface between the UNF beam helices and the ANC β-meander motif were targeted for site-directed mutagenesis to alanine or phenylalanine and introduced into virus genome for virus recovery by RG system (Fig. 6a; Table 2). From a structural perspective, none of these substitutions was expected to lead to change in the main-chain conformation or to interrupt steric or electrostatic effects.

**TABLE 2:** Mutations introduced to BTV1 VP5

Mutation	Reverse genetics	Virus growth in BSR cells	Recombinant protein
Unfurling domain			
H272A	Not recovered	NA^*a*^	ND^*b*^
H319A	Not recovered	NA	ND
Anchoring domain			
H365F	Not recovered	NA	ND
H384A	Recovered	Similar to WT	ND
H385A	Not recovered	NA	Still form trimer, loss of fusion activity
H386A	Recovered	Attenuated	ND
H412F	Not recovered	NA	ND
H465F	Recovered	Highly attenuated	ND

a NA, not applicable.

b ND, no data.

Mutations H272A and H319A within the UNF and H365F and H412F located in the ANC domain failed to generate any viable virus (Table 2). One mutation, H465F in the ANC, was recovered but was highly attenuated in that plaques appeared more slowly and were smaller than the wild-type virus (Fig. 6b), which was confirmed by a single-step growth curve of the recovered virus (Fig. 6c). Interestingly, as measured by virus recovery, while the mutation at H384 to alanine had no apparent effect on virus recovery, alanine substitutions of two consecutive His residues at H385 and H386 had significant effects on viral viability. Previously, using recombinant VP5 protein and synthetic liposomes, we showed that a triple (H384-6F) mutation, but not individual mutations, led to the complete loss of VP5 membrane penetration activity (1). In contrast to the recombinant protein data, H385A severely attenuated virus recovery. Similarly, mutation H386A also had an attenuated phenotype but less so, with smaller plaques and slower growth (Fig. 6b and c).

**FIG 7:**
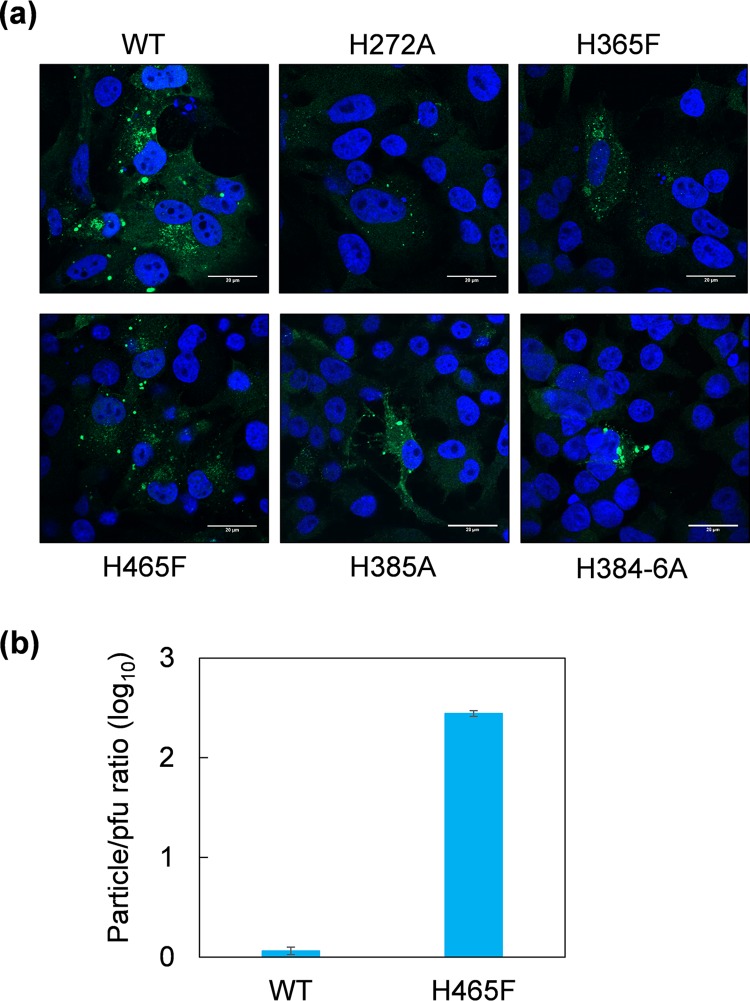
(a) Expression and localization of H272A, H365F, H465F, H385A, and H384-6A mutant VP5 proteins compared with that of the wild-type VP5 in BSR cells transfected with the capped mutant, or wild-type S5 RNA segments together with nine other BTV RNA segments were visualized by confocal immunofluorescence microscopy. Wild-type and mutant VP5 proteins are shown in green. Nuclei are shown in blue. (b) Average particle/PFU ratios of wild-type and H465F mutant viruses are 1.2 and 278.1, respectively.

To ensure that failure to recover H272A and H365F viruses or the highly attenuated H465F virus was not due to a disruption of mutant protein expression and that VP5 was still processed correctly in the expressing cells, BSR cells were transfected with the mutant RNA segments together with the other nine BTV RNA segments, and VP5 distribution was assessed by immunofluorescence. This confirmed that expression levels were comparable to the wild-type VP5 (Fig. 7a). When the same experiments were repeated with VP5 H385A and H384-6A, both mutant proteins were also visualized with identical distribution to the wild type, confirming that both mutants were equivalent to the wild type (Fig. 7a).

To examine if the mutation affected the particle to PFU ratios of the highly attenuated mutant viruses, we determined the number of particle versus PFU of one representative mutant virus, H465F, which showed an average particle/PFU ratio of approximately 278. This was significantly higher than that of the wild-type virus which had an average particle/PFU ratio of 1.2 (Fig. 7b), indicating that this mutation severely impaired the efficiency of infection. Together, these data demonstrate that a single substitution of histidine in certain locations has a profound effect on virus entry, suggesting it is not the cluster of His residues but their positions in VP5 that is key to function.

**FIG 8:**
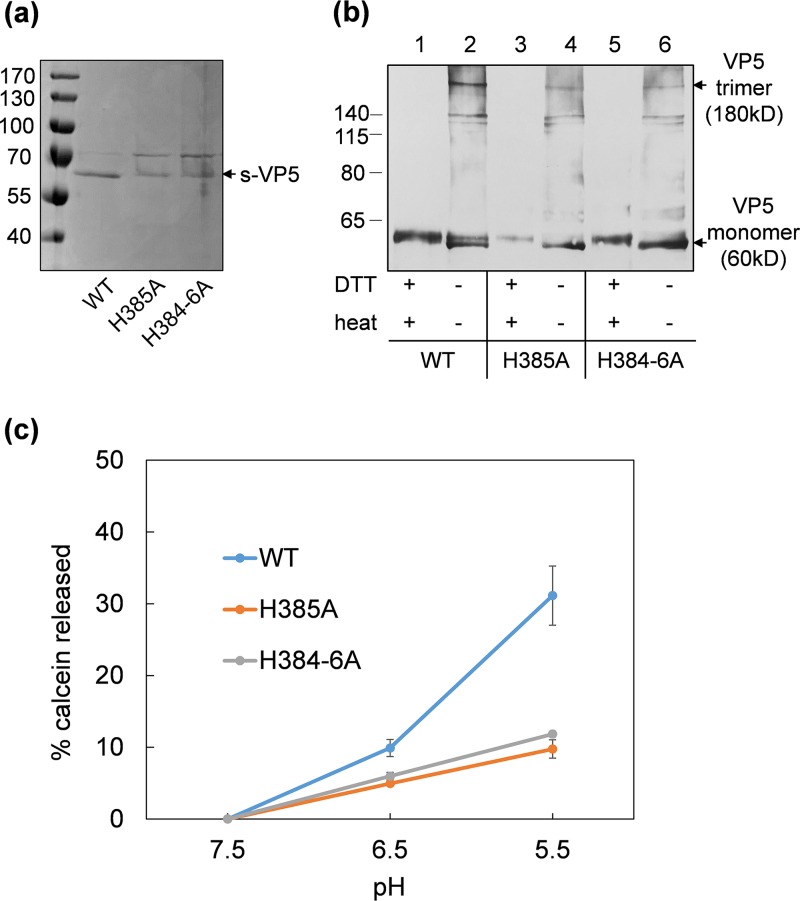
Analysis of recombinant VP5 mutant proteins. (a) Purified wild-type, H385A, and H384-6A mutant s-tagged VP5 proteins confirmed by SDS-PAGE with Coomassie blue staining. (b) Western blot using a VP5 antibody showing the monomer (60 kDa) and trimer (180 kDa) of wild-type and H385A, H384-6A mutant proteins in the absence of DTT reducing agent and 100°C heat treatment to the purified protein. Trimer and monomer account for about 25% and 75%, respectively, in nondenaturing samples (lanes 2, 4, and 6) compared with denaturing samples (lanes 1, 3, and 5) quantified by GeneTools (SynGene). (c) Late endosome membrane permeability of wild-type or H385A and H384-6A mutant proteins measured by calcein release from liposome mimicking late endosome membrane at neutral and acidic pH.

Further, to ensure H385A did not impact the overall structure of VP5, which might have caused failure of virus recovery, the mutant was expressed using the baculovirus expression system, and while protein expression was lower (Fig. 8a), the ability of the mutant protein to trimerize, a key measure of folding, was not affected (Fig. 8b). Furthermore, when the VP5 H385A and a triple mutant protein, H384-6A, were assessed for membrane-penetration activity using a liposome composed of lipids resembling the late endosome (6), both mutants failed to show any activity in contrast to the wild type but essentially similar to that of the triple (H384-6F) mutant described previously (1) (Fig. 8c).

### Determination of the anchoring domain role in the absence of the other VP5 domains.

**FIG 9:**
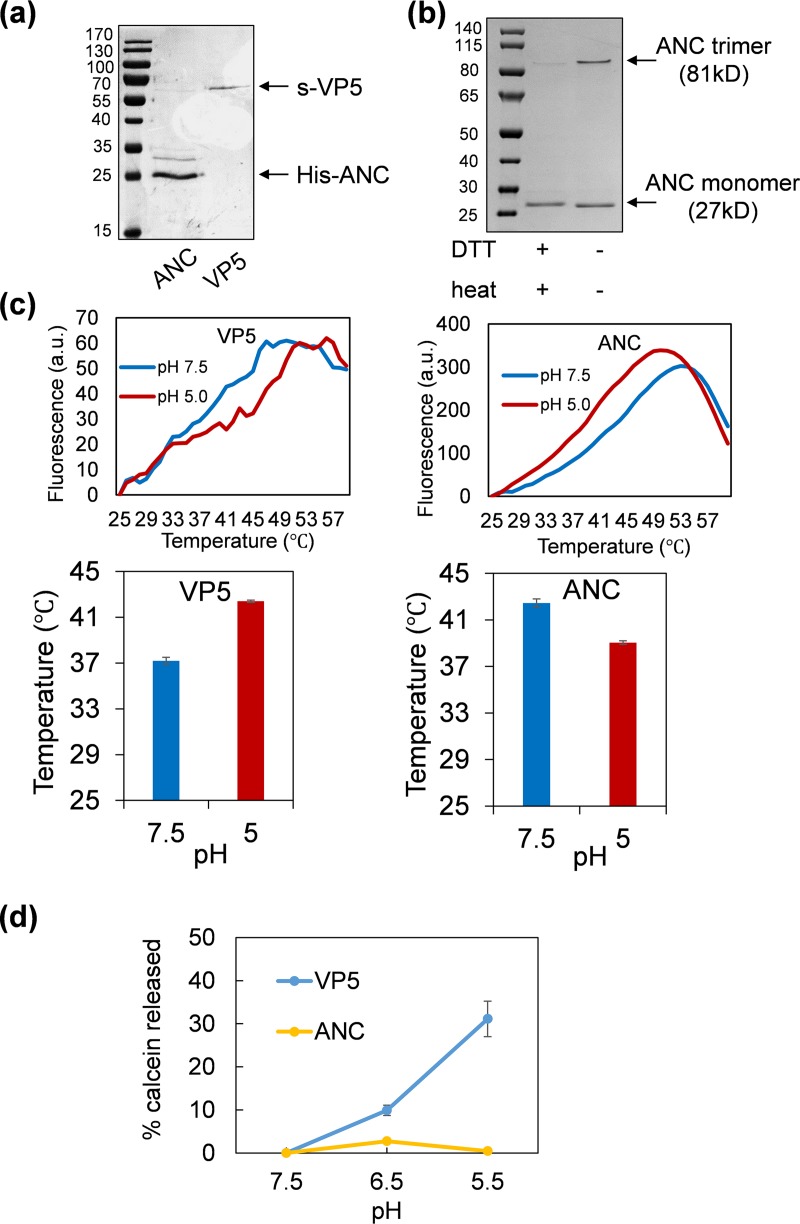
Analysis of recombinant ANC polypeptide. (a) Purified his-tagged ANC and wild-type s-tagged VP5 proteins confirmed by SDS-PAGE with Coomassie blue staining. (b) A 4 to 12% NuPAGE (Novex) Bis-Tris MOPS gel with Coomassie blue stating showing the trimeric (81 kDa) and monomeric (27 kDa) forms of purified His-tagged ANC in the presence or absence of DTT reducing agent and 100°C heat treatment. Trimer and monomer account for about 30% and 70%, respectively, quantified by GeneTools (SynGene). (c) The effect of pH on the stability of wild-type VP5 and ANC was measured, the melting temperature (*T_m_*) was compared and presented as histograms, and corresponding melting curves presented in the top panel. (d) Late endosome membrane permeability of wild-type VP5 and ANC proteins measured by calcein release from liposome mimicking late endosome membrane at neutral and acidic pH.

Imaging of the BTV particle in low pH by cryo-electron microscopy (cryo-EM) revealed that while the anchoring domain of VP5 remained attached to the underlying VP7 layer, both the dagger and unfurling domains refolded to form a flexible barb-like structure, potentially the membrane fusion form (1). To confirm the relative pH stability of this domain, it was expressed alone and its form was characterized in solution by gel electrophoresis (Fig. 9a). In the absence of the other two domains, the purified ANC domain retained its trimeric structure (Fig. 9b), consistent with the cryo-EM observations (1). Thermal shift assays were performed to determine if the recombinant ANC polypeptide itself could sense pH or if this was solely a property of the other two domains. We found that the isolated ANC polypeptide responded to late endosomal pH (5.0) but that its melting temperature decreased compared with the wild-type protein, suggesting a lower stability in the absence of the UNF and dagger domains (Fig. 9c). A pore formation assay confirmed that the ANC polypeptide alone was incapable of pore-forming activity (Fig. 9d). Together, these results suggest that the UNF and dagger domains of VP5 are the primary sensors of the late endosome and undergo significant conformational change, leading to membrane penetration. The ANC domain remains attached to VP7 long enough to drag the core particle through the disrupted membrane but with the UNF and dagger domains now removed, eventually drops the VP7 contact to release the core into the cytoplasm.

## DISCUSSION

The high-resolution cryo-EM structure has made it possible to deduce how the outer capsid proteins VP2 and VP5 of BTV coordinate the process of cell entry. A comprehensive structure-function analysis of residues in VP2 and VP5, predicted to be key to function, revealed a novel role for the unique zinc finger found in VP2 and certain residues within a His cluster found in VP5. These roles relate to the disengagement of VP2 from the complete virion in the early endosome followed by the activation of the now exposed VP5 in the late endosome. This stepwise and concerted entry mechanism requires the ability to accurately sense the relative pH at each location. Despite apparently favorable locations at the interface of the VP2 hub domain with the top of VP7, no role was found for histidine in this location. This is somewhat surprising, as H866 is conserved across all BTV serotypes and had been previously proposed to play a role in the entry process (1). Similarly, H95, located in a loop that interacts with VP5, conserved H38, H900, and H925 in the hub domain, and H426, H640, and H756 in the body domain were found not to play a key role in pH sensing. In contrast, H164, part of a tetrahedral zinc-finger motif together with C162, C617, and C851, was essential for virus recovery; importantly, mutagenesis of any of these residues was lethal but not due to a disruption of protein expression and integrity. Our data demonstrated that both the conformation of the finger and the correct position of histidine were critical for the zinc finger to be functional during virus entry. The recombinant protein bearing C162H lost its ability to bind zinc and to sense low pH, although it is still capable of attaching to cells for entry, as demonstrated by hemagglutination activity, providing a direct link between the function of the zinc finger and the function of VP2 as a whole. We are not aware of any precedent for a zinc finger acting in virus entry in this way. Of the few previous examples, a dual zinc-finger structure in the G1 envelope glycoprotein of bunyavirus was proposed to be involved in protein-protein or protein-RNA interactions (13, 14), and a zinc-binding domain (ZBD) found in Junin virus (15, 16) was proposed to modulate pH-dependent membrane fusion. However, neither of these mechanisms directly predicts our discovery of this zinc finger action in VP2.

Our analysis also revealed how VP5 senses the late endosomal pH to trigger membrane permeabilization. VP5 contains a total of 19 His residues, 13 of which are clustered at the interface of the anchoring domain and the helices of the unfurling domain. This His cluster previously speculated as functional within the ANC domain was investigated. Most of the His residues in the ANC domain rendered the virus inviable or attenuated although the expression and integrity of VP5 are not significantly affected when mutated singly, indicating the location of each histidine might be more important than the cluster itself, as previously speculated (1). Notably, three consecutive H384, H385, and H386 residues had dramatically different effects, from lethal to none, when mutated individually. Structural analysis revealed that H384 has more space and neighboring charged residues are pointing away. In contrast, in the other two cases, H385 and H386, the pocket is tight and charged residues directly point at the His residues. This indicated that the interaction between H385-H386 and neighboring charged residues is important for maintaining the VP5 conformation and function. Surprisingly, the distinct effects observed for each single substitution mutant could be delineated for their precise positions in VP5. Those that exhibited a lethal effect are likely to be responsible to initiate the conformational change in response to low pH protonation due to their more exposed positions. The ANC domain itself was shown to be sensitive to low pH but was stable enough to maintain a tertiary structure necessary for VP5 function overall. VP5 has been proposed to share structural features with class I viral fusion proteins of enveloped viruses (1, 9, 17), and experimental evidence has been obtained for a “fold-back” model of action, akin to the type I fusion proteins, and for the rotavirus VP5 protein (18). It seems plausible that BTV VP5 may use a similar strategy, although the fine detail remains to be determined.

Our comprehensive molecular analysis reveals key amino acids of VP2 and VP5 required for detecting cellular pH, leading to an irreversible change that propels the virus through the cell membrane. Our data illustrate a novel function of zinc finger in sensing pH and identify for the first time the key residues essential for cell entry by BTV. Surprisingly, the amino acid residues that we identified as essential for BTV entry lie outside those predicted previously based on the virus structure. Our data further highlight the dynamic nature of the multiconformational process required for virus infection and indicate a potential mechanism that may be shared by other similar viruses, and may be targets for future therapies.

## MATERIALS AND METHODS

### Site-directed mutagenesis.

Site-directed mutagenesis, as previously described (1), was performed to introduce mutations into the exact copy of BTV-1 S2 or S5 (pUCBTV1T7S2 or pUCBTV1T7S5) sequences, for reverse genetics, or into baculovirus transfer vectors, pAcYM1-S·tag-BTV1VP2 and pAcYM1-S·tag-BTV1VP5, for protein expression.

### Single-step viral growth curve.

BSR cells were infected with wild-type or mutant virus at a multiplicity of infection (MOI) of 0.1 for 0, 24, 48, and 72 h in triplicate. Supernatant virus was collected at each time point and the titers were determined by plaque assays to generate the growth curve.

### Recombinant protein expression.

Wild-type or mutant VP2 and VP5 proteins were expressed by infecting sf9 cells at an MOI of 5 with recombinant baculovirus for 48 h. Cells were lysed in the lysis buffer (50 mM Tris-HCl [pH 8.0], 200 mM NaCl, 1 mM EDTA, 1% NP40, and protease inhibitor cocktail), and the S-tagged protein was purified using the S-protein agarose (Merck Millipore) and eluted with 3 M MgCl_2_. It was then desalted with a PD-10 desalting column (GE Healthcare). VP5 ANC (P348-A526) in the pET28 backbone was expressed in BL21(DE3) pLysS cells (Invitrogen) induced with 1 mM isopropyl-β-d-thiogalactopyranoside (IPTG) for 4 h. Cells were lysed in lysis buffer (50 mM NaH_2_PO_4_ [pH 8.0], 300 mM NaCl, 5 mM imidazole, 1% NP40, and protease inhibitor cocktail), and the lysate was applied to cobalt resin (Sigma) for His-tagged protein purification.

### Thermal shift assay.

A total of 20 μl of purified protein at 0.2 mg/ml was mixed with 5× SYPRO orange (Invitrogen). For the metal-binding experiments, VP2 was either untreated or incubated with Chelex-100 resin (Bio-Rad) and 1× DTT for 1 h. For the pH experiments, protein was acidified to the stated pH with 0.1 N HCl. The assay was performed on an MX3005P q-RT PCR system (Agilent Technologies) with the temperature ramped from 25 to 95°C at 45 s/°C as previously described (1).

### Analysis of VP5 oligomerization by Western blot.

The multimeric nature of wild-type and mutant VP5 was analyzed by 10% NuPAGE morpholinepropanesulfonic acid (MOPS) SDS gel (Invitrogen) using a guinea pig anti-VP5 antibody. The protein was prepared in NuPAGE lithium dodecyl sulfate (LDS) sample buffer (Thermo Fisher) with or without DTT and with or without heating at 100°C for 5 min. The bands were analyzed using GeneTools (SynGene) to determine the monomeric and trimeric VP5.

### Immunofluorescence.

A BSR monolayer transfected with 1 μg of wild-type or mutant S5 together with nine other BTV RNA segments using EndoFectin according to manufacturer’s instruction (GeneCopoeia) was fixed in 4% paraformaldehyde and then permeabilized with 0.1% Triton X-100 in phosphate-buffered saline (PBS). The guinea pig anti-VP5 antibody was used to detect VP5, using the relevant secondary fluorescent antibody. Nuclei were stained with Hoechst. Images were captured using the LSM510 inverted confocal microscope (Carl Zeiss Ltd.).

### Liposome calcein release assay.

Five micrograms of the total lipid with a ratio of 13:5:1:4 PC/PE/PS/BMP (Sigma) in 1 ml of the calcein buffer (50 mM calcein, 100 mM NaCl, 10 mM Na_2_HPO_4_, and 2 mM KH_2_PO_4_) was prepared using a mini extruder with a 0.1-μm polycarbonate membrane (Avanti Polar Lipids). Unencapsulated calcein was removed by size exclusion chromatography. Purified WT or mutant VP5 or ANC was mixed with calcein-loaded liposomes at a final concentration of 0.1 mg/ml and incubated at room temperature for 10 min. The mixture was then acidified to the stated pH with 0.1 M HCl and incubated for 20 min at 37°C. Fluorescence was measured, and the percentage of calcein release was calculated as previously described (1).

### Hemagglutination assay.

A total of 25 μl of 2-fold serial dilutions of 0.5 mg/ml purified wild-type and C162H mutant VP2 proteins were mixed with 50 μl of 0.25% washed sheep erythrocytes (Thermo Fisher Oxoid Ltd.) in U-bottom 96-well plate and incubated for 1 h at room temperature as previously described (3). Then, the hemagglutination effects were observed. PBS dilution buffer was used as negative control.

### Quantitative RT-PCR.

Viral RNA was extracted using the GeneJET viral DNA and RNA purification kit (Thermo Fisher) following the manufacturer’s instruction. A total of 10 μl of eluent was used for cDNA synthesis using the RevertAid reverse transcriptase (Thermo Fisher) with a BTV S6 (NS1)-specific primer (GTAAGTTGAAAAGTTCTAGTAG). A total of 1 μl of 1:5 diluted cDNA was then used for quantitative PCR (qPCR) using the Maxima SYBR Green/ROX qPCR 2× master mix (Thermo Fisher) with primers (forward [Fw], GGACGATACCGGATTGGAATAA; reverse [Rw], CATCGTAGCATAAGCCCTCTC) targeting the S6, following the manufacturer’s instruction. The viral particle number was estimated on the basis of a viral genome copy number determined by qRT-PCR.

## Supplementary Material

Supplemental file 1
