# Supplementary material for: Mapping the pH Sensors Critical for Host Cell Entry by a Complex Nonenveloped Virus
Source: J Virol. 2019 Feb 5;93(4):e01897-18. doi: 10.1128/JVI.01897-18 (PMC6363992; doi:10.1128/JVI.01897-18)
Supplement: Supplemental file 1 [file c59d2f62afdfc5f70016630517ed09cc_JVI.01897-18-s0001.pdf]

Supplementary figure S1 Alignment of VP2 sequences (searched in Genebank) from different BTV serotypes by Clustal Omega online tool. Highly conserved His residues highlighted in red boxes.

Supplementary figure S2 Alignment of VP5 sequences (searched in Genebank) from different BTV serotypes by Clustal Omega online tool. Highly conserved His residues highlighted in red boxes.

Supplementary Fig. S1

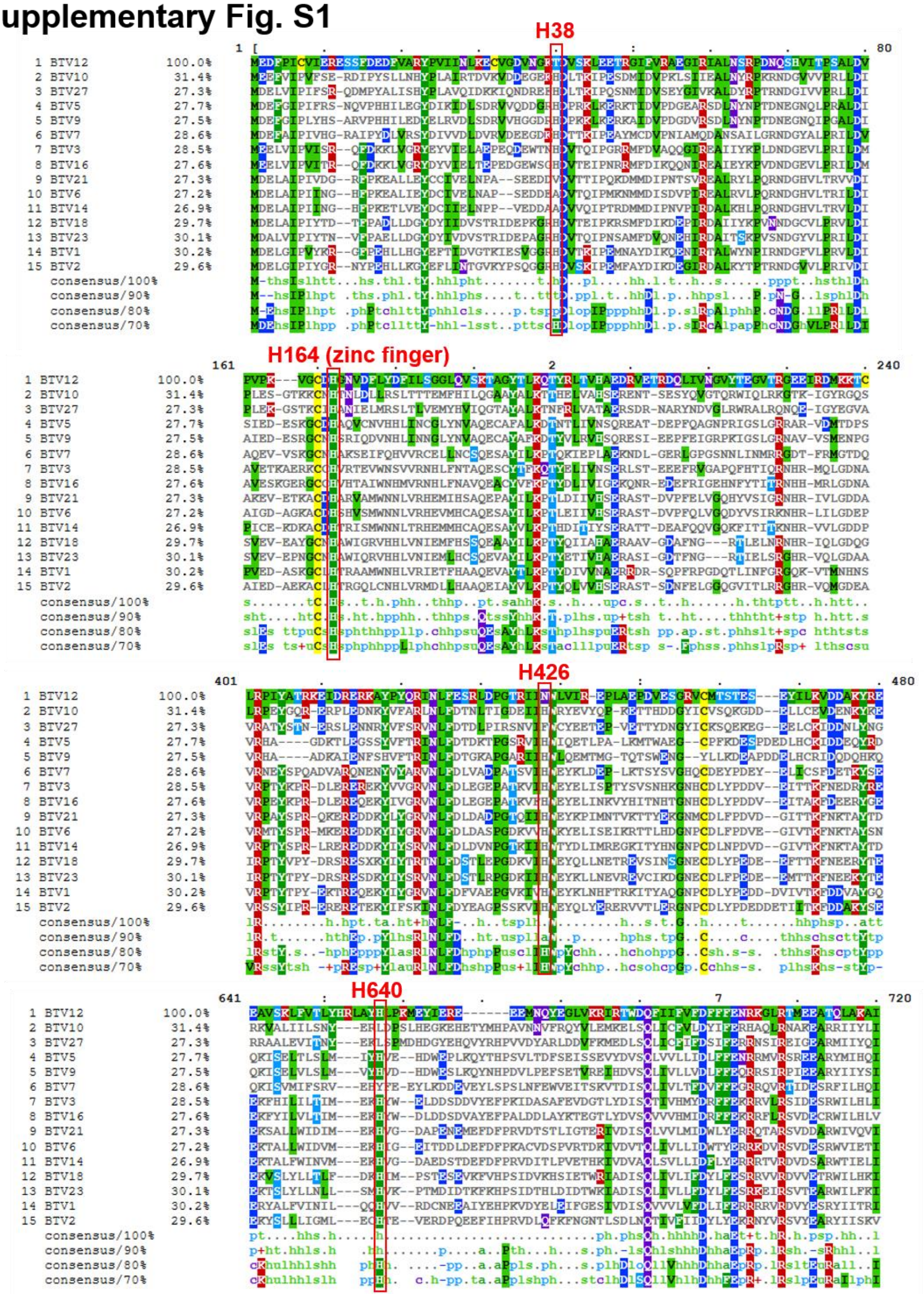

Continued

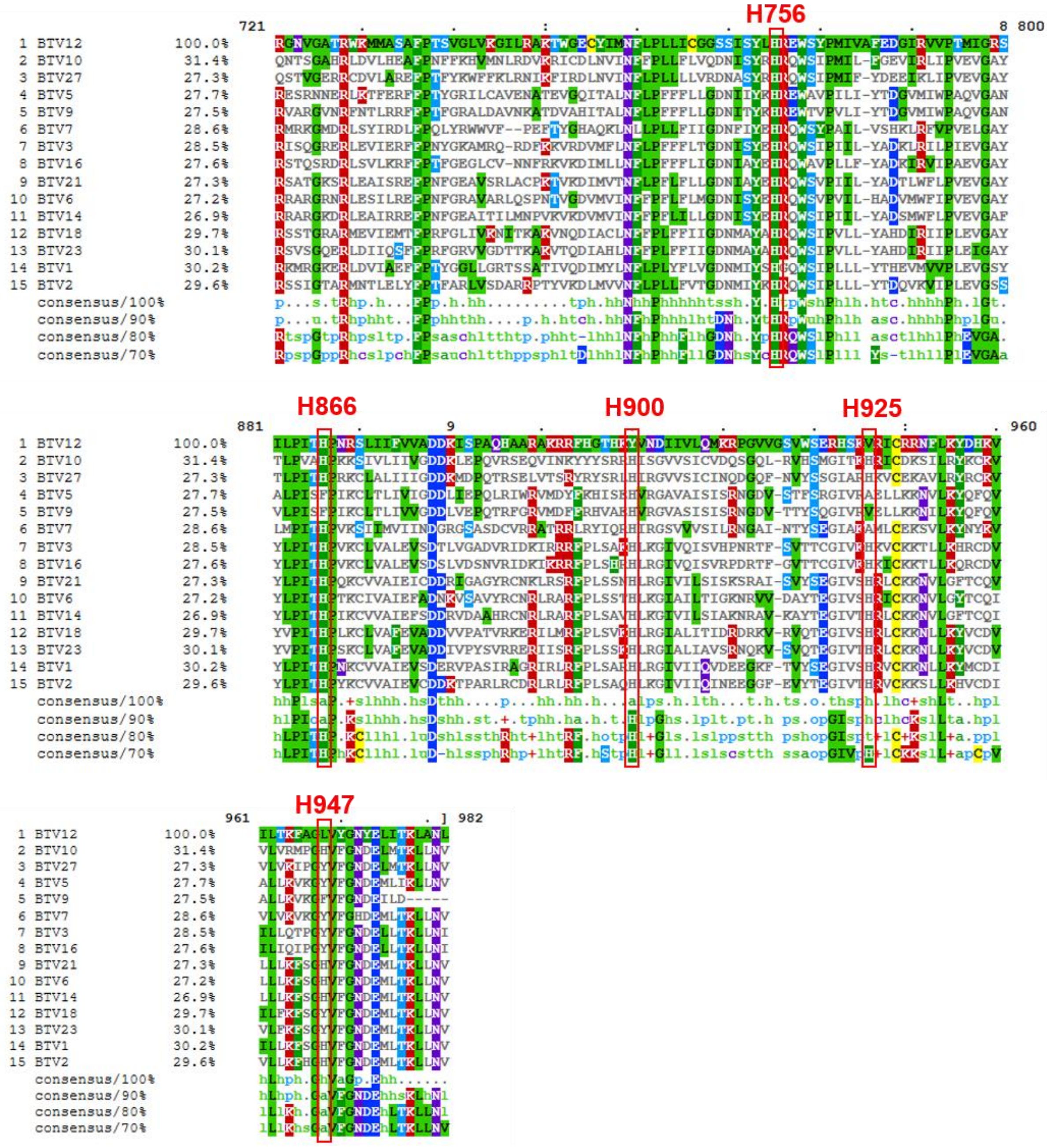
H756
H866
H900
H925
H947

Supplementary Fig. S2

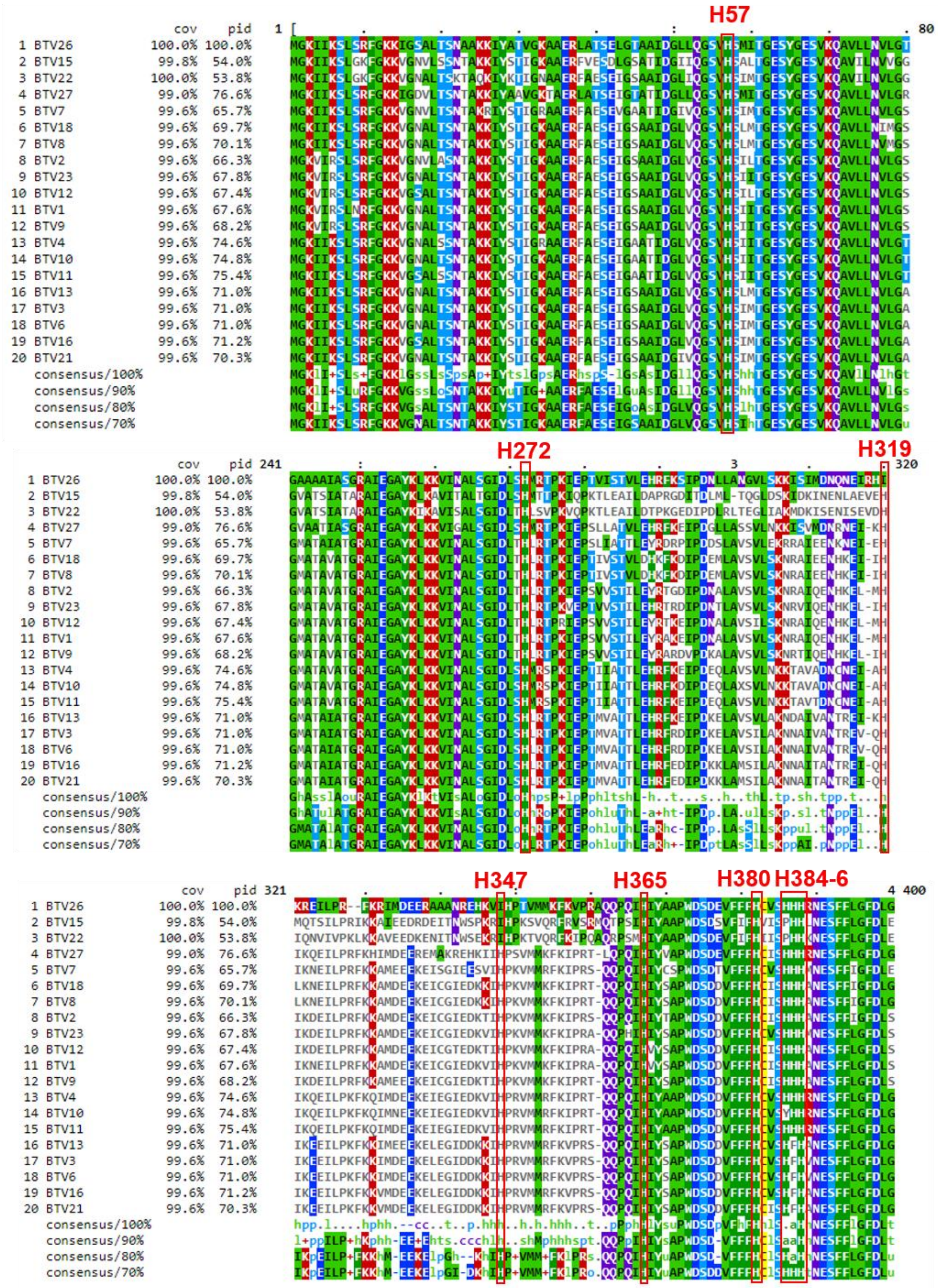

Continued

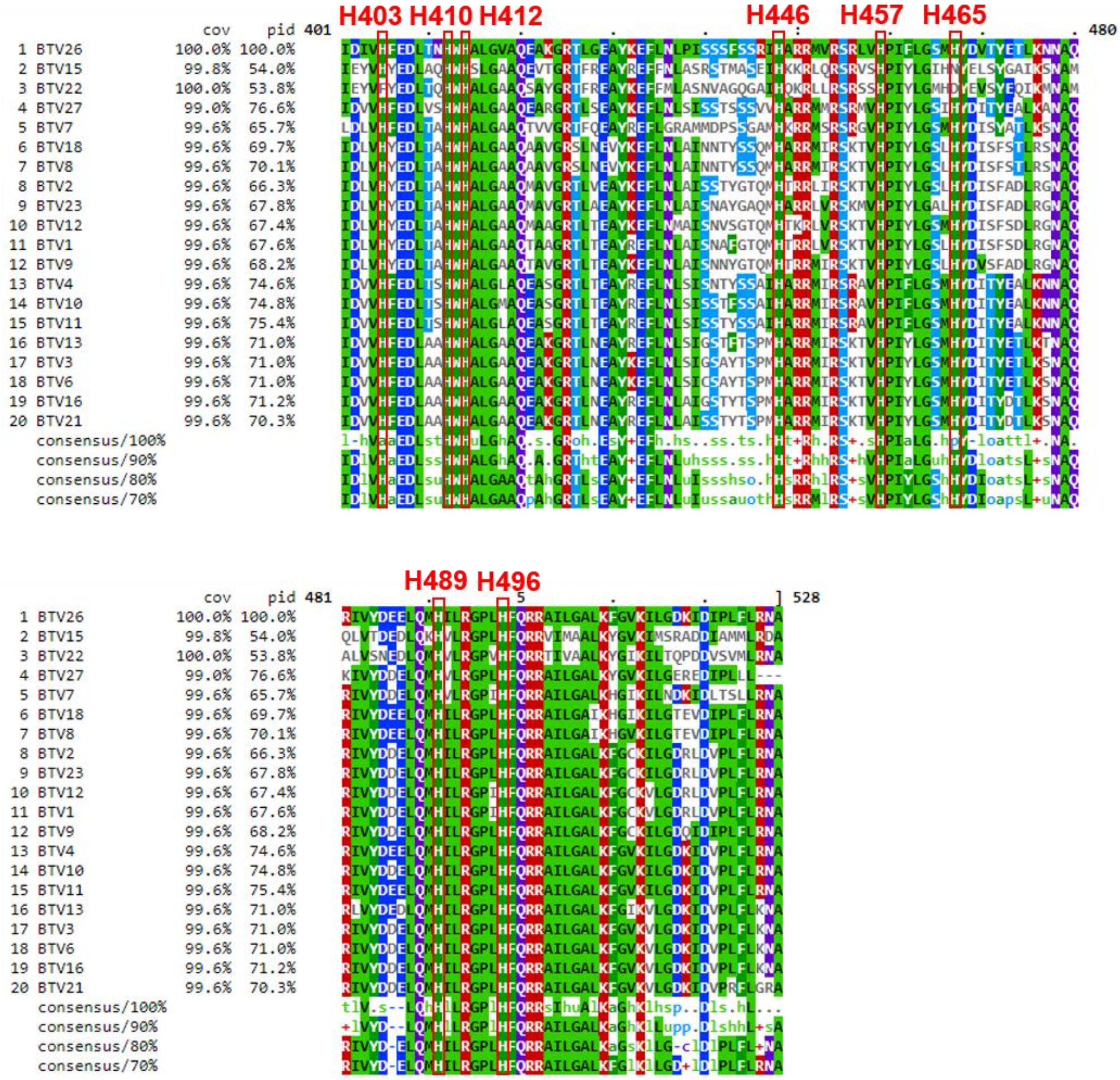
H403 H410 H412 H446 H457 H465
cov pid 401
480
1 BTV26 100.0% 100.0%
2 BTV15 99.8% 54.0%
3 BTV22 100.0% 53.8%
4 BTV27 99.0% 76.6%
5 BTV7 99.6% 65.7%
6 BTV18 99.6% 69.7%
7 BTV8 99.6% 70.1%
8 BTV2 99.6% 66.3%
9 BTV23 99.6% 67.8%
10 BTV12 99.6% 67.4%
11 BTV1 99.6% 67.6%
12 BTV9 99.6% 68.2%
13 BTV4 99.6% 74.6%
14 BTV10 99.6% 74.8%
15 BTV11 99.6% 75.4%
16 BTV13 99.6% 71.0%
17 BTV3 99.6% 71.0%
18 BTV6 99.6% 71.0%
19 BTV16 99.6% 71.2%
20 BTV21 99.6% 70.3%
consensus/100%
consensus/90%
consensus/80%
consensus/70%
H489 H496
cov pid 481
528
